# Chronic Administration of COVID-19 Drugs Fluvoxamine and Lopinavir Shortens Action Potential Duration by Inhibiting the Human Ether‐à‐go‐go–Related Gene and Cav1.2

**DOI:** 10.3389/fphar.2022.889713

**Published:** 2022-07-07

**Authors:** Zequn Zheng, Dihui Cai, Yin Fu, Ying Wang, Yongfei Song, Jiangfang Lian

**Affiliations:** ^1^ Department of Cardiovascular, Lihuili Hospital Facilitated to Ningbo University, Ningbo University, Ningbo, China; ^2^ Ningbo Institute of Innovation for Combined Medicine and Engineering, Ningbo, China; ^3^ Department of Cardiovascular Medicine, First Affiliated Hospital of Shantou University Medical College, Shantou, China

**Keywords:** fluvoxamine, lopinavir, novel coronavirus disease of 2019 (COVID-19), hERG potassium channel, CACNA1C, long QT syndrome

## Abstract

**Background:** Old drugs for new indications in the novel coronavirus disease of 2019 (COVID-19) pandemic have raised concerns regarding cardiotoxicity, especially the development of drug-induced QT prolongation. The acute blocking of the cardiac hERG potassium channel is conventionally thought to be the primary mechanism of QT prolongation induced by COVID-19 drugs fluvoxamine (FLV) and lopinavir (LPV). The chronic impact of these medications on the hERG expression has yet to be determined.

**Methods:** To investigate the effect of long-term incubation of FLV and LPV on the hERG channel, we used electrophysiological assays and molecular experiments, such as Western blot, RT-qPCR, and immunofluorescence, in HEK-293 cells stably expressing hERG and human-induced pluripotent stem cell–derived cardiomyocytes (hiPSC-CMs).

**Results:** Compared to the acute effects, chronic incubation for FLV and LPV generated much lower half-maximal inhibitory concentration (IC50) values, along with a left-shifted activation curve and retarded channel activation. Inconsistent with the reduction in current, we unexpectedly found that the chronic effects of drugs promoted the maturation of hERG proteins, accompanied by the high expression of Hsp70 and low expression of Hsp90. Targeting Hsp70 using siRNA was able to reverse the effects of these drugs on hERG proteins. In addition, FLV and LPV resulted in a significant reduction of APD90 and triggered the early after-depolarizations (EADs), as well as inhibited the protein level of the L-type voltage–operated calcium channel (L-VOCC) in hiPSC-CMs.

**Conclusion:** Chronic incubation with FLV and LPV produced more severe channel-blocking effects and contributed to altered channel gating and shortened action potential duration by inhibiting hERG and Cav1.2.

## Introduction

The pandemic of the novel coronavirus disease of 2019 (COVID-19), caused by the severe acute respiratory syndrome coronavirus 2 (SARS-CoV-2), poses an unprecedented healthcare challenge. Early in the epidemic, limited treatment options prompted many old drugs to enter the fight against SARS-CoV-2, including antimalarials chloroquine and hydroxychloroquine, macrolide antibiotics azithromycin, and antivirals lopinavir/ritonavir (LPV/r), and remdesivir. In a double-blind randomized controlled trial (RCT), fluvoxamine (FLV), a selective-serotonin reuptake inhibitor (SRI) to treat mental disorders was shown to have a lower clinical deterioration rate in hospitalized patients with COVID-19 (Lenze et al., 2020). Similarly, LPV/r affected some secondary patient outcomes such as a shorter time stay in the intensive care unit (ICU) (Cao et al., 2020a; Cao et al., 2020b). Importantly, in the absence of the observed reductions in viral load and large-scale RCTs, increased uncertainty arises after some studies with small sample sizes and low quality (Qaseem et al., 2020). Furthermore, given the fact that many drugs cause cardiotoxicity, particularly drug-induced QT prolongation, researchers have raised concerns about prescribing these drugs before large-scale RCTs are completed (Naksuk et al., 2020).

Drug-induced QT prolongation is an inevitable safety concern before they enter the clinical application, and drugs used in COVID-19 are no exception (Naksuk et al., 2020; Haghjoo et al., 2021). QT interval prolongation has also been reported in LPV/r-treated COVID-19 patients, and FLV has been classed as a drug that prolongs the QT interval although it is considered to have a lower risk relative to homogeneous SRIs (Yamazaki-Hashimoto et al., 2015; Ojero-Senard et al., 2017; Haghjoo et al., 2021). The reduction in the rapid activation of delayed rectifier potassium currents (I_Kr_), generated by the cardiac hERG potassium channel encoded by *KCNH2*, is widely thought to be primarily responsible for drug-induced QT prolongation, in which cardiotoxic drugs directly bind to high-affinity regions in the channel pore domain and/or interfere with the production of mature hERG proteins (Zequn et al., 2021). An integrated risk assessment for drug-induced QT prolongation for a series of repurposed drugs for COVID-19 and the acute inhibition of the hERG channel by FLV and LPV in a concentration-dependent manner has been well explored previously (Milnes et al., 2003; Anson et al., 2005; Michaud et al., 2021). Amino acid–directed mutagenesis studies have defined Y652 and F656 as key drug binding sites in the hERG channel, and mutations of them can partially ameliorate the blocking effect for FLV (Milnes et al., 2003). These studies treated tool cell HEK-293 with different concentrations of medications to detect acute inhibition of hERG currents and designated the FLV and LPV as cardiotoxic drugs that target the hERG potassium channel. *In silico* analysis of the LPV to hERG channel, F656 and Y652 in the central cavity revealed that they can interact with the medication in the low energy state (Al-Moubarak et al., 2021). The acute binding of these key sites by multiple groups of drugs does not alter the number of hERG channels on the cell membrane but rather affects their ion permeation or gating kinetics.

Critically, a growing number of drugs have been recognized to disrupt the hERG proteins’ biosynthesis to cause the reduction of current, a process requiring numerous molecular chaperones, such as the critical heat shock family of Hsp70 and Hsp90. These molecular chaperones bind the hERG protein’s core-glycosylated form (immature form) to drive it away from the endoplasmic reticulum for the following full-glycosylation (mature form), demonstrating their fundamental position in the maturation of hERG (Ficker et al., 2003; Nogawa and Kawai, 2014). Drugs that reduce the expression of the hERG protein require long-term immersion of cells expressing the hERG channel in the external solution containing a series of concentrations of drugs, a chronic process different from the transient effect of acute drug addition and elution to detect the hERG current. Of note, COVID-19 patients are often prescribed multi-day regimens for these drugs, and it remains unclear whether the long-term administration for them will cause a reduction in hERG maturation and involve changes in the expression of molecular chaperones.

To determine the chronic effects of COVID-19 drugs FLV and LPV on cardiac hERG potassium channels, we performed long-term incubations of these drugs with HEK-293 cells stably expressing hERG and human-induced pluripotent stem cell–derived cardiomyocytes (hiPSC-CMs). We used electrophysiological analysis to determine the half-maximal inhibitory concentration (IC50) of chronic hERG inhibition, as well as changes in channel activation gating and action potential duration (APD) to assess the drug’s toxicity potential incubated for at least 24 h. Furthermore, the effects of drugs in more ion channels, particularly the L-type voltage–operated calcium channel (L-VOCC), are also characterized here. Molecular evidence will be used to disclose the expression of the hERG protein and its key mature chaperones Hsp70 and Hsp90 to evaluate the effects of FLV and LPV on hERG trafficking.

## Materials and Methods

### Molecular Biology

HEK-293 cells stably expressing the hERG protein (HEK-hERG) and hiPSC-CMs were used to evaluate the chronic effects of 24 h incubation with the drug on hERG channels. The HEK-293 cell line stably expressing hERG was created using plasmid transfection followed by puromycin (2 μg/ml) selection. HEK-hERG was cultured in DMEM (high glucose) containing 10% FBS (Gibco, United States) and kept in a humid 5% CO_2_ incubator at 37°C.

### Generation and Characterization of Cardiomyocytes From Human-Induced Pluripotent Stem Cells

A hiPSC line was purchased from the Cell Bank of the Chinese Academy of Sciences (SCSP-1301; Shanghai, China). The continuous cultures reached a steady state after 20 generations in the Pluripotency Growth Master 1 (PGM1 culture system) (CA1007500; Cellapy, Beijing, China). Generation of cardiomyocytes from hiPSC was performed by following the previously reported method with modifications (Jiang et al., 2018). For the differentiation of hiPSC into cardiomyocytes, a cardioeasy chemically defined cardiac differentiation kit (CA2004500; Cellapy, Beijing, China) was used. Briefly, hiPSCs were isolated using the Gentle Cell Dissociation Reagent (100–0,485; STEMCELL Technologies, Vancouver, Canada) at a 1:4 ratio and cultured in a 6-well plate. When reached ∼80% confluence, hiPSCs were cultured in induction medium I for 2 days and then in induction medium II for another 2 days. Induction medium III added with 10 μM KY02111 (S7096; Selleck, Shanghai, China) was changed every 48 h until the spontaneous beating of cells was observed. Cardioeasy maintenance and the purification medium (CA2015002 and CA2005100; Cellapy, Beijing, China) were used for long-term culture and purification of hiPSC-CMs on day 15 after induction.

### Patch-Clamp Electrophysiological Recordings

For the evaluation of chronic inhibition, the HEK-hERG cells and hiPSC-CMs were collected from the culture dish to be resuspended into single cells and kept for up to 4 h in the medium at room temperature until use. The hERG current (I_hERG_) in HEK-hERG cells and action potentials (APs) in hiPSC-CMs were recorded using a whole-cell patch-clamp system in the voltage-clamp and current-clamp mode, respectively. Compounds were perfused with a range of concentrations (0.5 μM–30 μM) during patch-clamp recordings while bathed using the slice perfusion system to determine the respective acute inhibitory effects. Pipettes were pulled from a thin-walled borosilicate glass using a micropipette puller (P-1000, Sutter Instrument, Novato, CA) to achieve a resistance of 2.0–6.0 MΩ. With a series resistance compensation of 80%, the capacitance and resistance of the whole cell were adjusted. MultiClamp 700 B amplifier, Digidata 1550 B digitizer, and pCLAMP10.3 (Axon Instruments) were used for data amplification, acquisition, and analysis, respectively. After the medium was discarded, cells were superfused with the bath solution containing (in mM) 4 KCl, 137 NaCl, 1.8 CaCl_2_, 10 glucose, 1 MgSO_4_, and 10 HEPES (pH7.4 with NaOH). The pipette solution contained (in mM) 130 KCl, 5 EGTA, 5 MgATP, 1 MgCl_2_, and 10 HEPES (pH 7.2 with KOH).

Step currents were elicited by 3 s of depolarizing steps from a holding potential of −80 mV to potentials ranging from −60 mV to +50 mV in 10 mV increments. This was followed by a 3 s repolarization phase to −40 mV to elicit tail currents. After the compensation of membrane capacitance and series resistance, spontaneous APs were recorded in a gap-free mode and were paced with depolarizing 20 ms-pulses from 0 to 300 pA by 20 pA increments.

### Western Blot Analysis and Protein Knockdown Using Small Interfering RNA

Following 24 h of drug incubation, HEK-hERG cells and hiPSC-CMs were lysed in RIPA buffer supplemented with 1 mM PMSF (phenylmethylsulfonyl fluoride) and 4% protease inhibitor cocktail (100:1, APExBIO Technology LLC, Houston, United States). Protein concentrations were estimated using the Pierce BCA protein Assay kit following the manufacturer’s instructions (DQ111-01; TransGen, Beijing, China).

Equal amounts of protein (20 µg) were loaded on 7.5% SDS polyacrylamide electrophoresis gels for separation at 100 V for 1.5 h. Separated proteins were transferred onto polyvinylidene difluoride (PVDF) membranes at a constant current of 200 mA for 2.5 h in a cold room (4°C). The membranes were blocked for 2 h using 5% non-fat milk in TBST (Tris-buffered Saline with 1% Tween 20) and then were incubated overnight with the primary antibodies. The following day, PVDF membranes were incubated with the corresponding horseradish peroxidase (HRP)-–conjugated secondary antibodies in TBST for 1 hour. Primary and secondary antibodies were washed using TBST for 10 min every four times. The blots were visualized with ImageQuant LAS 500 (GE, JPN) using an enhanced WesternBright ECL chemiluminescent substrate (Advansta, United States). Protein band densities were determined using ImageJ (United States) and values were normalized with β-tubulin as the loading control.

Sequences of siRNAs targeting HSPA1A were chemically synthesized from Shanghai Gene Pharma. Equal doses (100 pmol) of target siRNA or negative control siRNA using 10 μl Lipofectamine RNAi MAX reagents (Invitrogen, Carlsbad, CA) in Opti-MEM (Gibco) were transfected into cells for 48 h and the knock-down efficiency was verified by immunoblotting. The desirable sequences are as follows: siHsp70: 5′-GCA​ACG​UGC​UCA​UCU​UUG​ATT-3′; negative control: 5′-UUC​UCC​GAA​CGU​GUC​ACG​UTT-3′.

### Quantitative Real-Time Reverse Transcription PCR

Total RNA for hiPSC-CMs was isolated using the EasyPure RNA Kit following the manual (ER101, TransGen, Beijing, China). A measure of 1 μg of RNA templates for each sample was reverse-transcribed using TransScript All-in-One First-Strand cDNA Synthesis SuperMix (AT341, TransGen Biotech, China) according to the manufacturer’s instructions. Real-time PCR was performed in triplicate with Taq Pro Universal SYBR qPCR Master Mix (Q712, Vazyme, Nanjing, China) and specific primers. The samples were then cycled in an Applied Biosystems 7,500 real-time PCR system as follows: 2 min at 95°C, followed by 40 cycles of 10 s at 95°C, and 1min at 65°C. Relative quantification was calculated using the 2^−ΔΔCq^ method relative to the housekeeping gene GAPDH. The following are the prime sequences: *SLC8A1*: ACA​ACA​TGC​GGC​GAT​TAA​GTC (forward primer) and GCT​CTA​GCA​ATT​TTG​TCC​CCA (reverse primer), *CACNA1C*: TGA​TTC​CAA​CGC​CAC​CAA​TTC (forward primer) and GAG​GAG​TCC​ATA​GGC​GAT​TAC​T (reverse primer), *SCN5A*: TCT​CTA​TGG​CAA​TCC​ACC​CCA (forward primer) and GAG​GAC​ATA​CAA​GGC​GTT​GGT (reverse primer), *KCNE2*: AGA​AGA​GAG​CTC​GCT​AAC​GC (forward primer) and ACA​TGC​TTC​CCT​CCT​GCT​ATG (reverse primer), *KCNQ1*: CGC​GTC​TCC​ATC​TAC​AGC​A (forward primer) and GGA​CGA​TGA​GGA​AGA​CGG​C (reverse primer), *GAPDH*: GTT​CGT​CAT​GGG​TGT​GAA​CC (forward primer) and GCA​TGG​ACT​GTG​GTC​ATG​AGT (reverse primer).

### Immunofluorescence and Confocal Imaging

The HEK-hERG cells and hiPSC-CMs were fixed with 4% PFA, permeabilized with 0.3% Triton X-100, blocked with 5% BSA, and incubated overnight with the primary antibody for the hERG. The following day, the primary antibody was washed and the cells were incubated with the anti-mouse CoraLite488–conjugated secondary antibody and Alexa555-conjugated phalloidin for 1 hour, and then counterstained with DAPI. For immunocytochemistry of hiPSC-CMs, rabbit anti-troponin T (15513-1-AP; Proteintech, Wuhan, China) and mouse anti-sarcomeric α-actinin (66895-1-Ig; Proteintech, Wuhan, China) were used as primary antibodies. Anti-mouse CoraLite594 and anti-rabbit CoraLite488–conjugated secondary antibodies were used for immunofluorescence detection. The slides were examined under a Leica TCS SP8 confocal laser scanning microscope (Leica Microsystems, Inc.). Image processing and quantification were performed using ImageJ software. The density values of the three areas of each slide of each group are extracted using the profile analysis in the software, and then the density mean is used to generate a line scan image using the GraphPad Prism. At least 2 cell climbing slices from each group were replicated.

### Drugs and Reagents

FLV and LPV were purchased from APExBIO Technology LLC (B1205 and A8204; Houston, United States). Both drugs were diluted in DMSO to stock solutions of 10 mM, and stored at −20°C. To study blocking effects, the drugs were diluted to 2 mM in DMSO. The rabbit anti-hERG primary polyclonal antibody was purchased from Alomone Labs (APC-062; Jerusalem 9,104,201, Israel) and was used in a 1:400 dilution. The rabbit anti-Hsp90 primary polyclonal antibody and mouse anti-Hsp70 primary polyclonal antibody were purchased from Abcam (ab203126 and ab2787; Shanghai, China). The rabbit anti-CACNA1C primary polyclonal antibody for Western blotting was obtained from Affinity BioSciences (DF2267, Jiangsu, China) with a dilution of 1:500. The rabbit anti-L-VOCC primary polyclonal antibody for immunofluorescence was obtained from Proteintech (21774-1-AP, Wuhan, China) with a dilution of 1:200. The anti-Hsp90 primary antibody and anti-Hsp70 primary antibody were used in a 1:10,000 and 1:1,000 dilution, respectively. Anti-β-tubulin primary antibody and HRP- conjugated secondary antibody were purchased from TransGen Biotech (HC101, HS201-1, and HS101-1; Beijing, China), and they were both used in a 1:3,000 dilution. The fluorescent secondary antibodies were purchased from Proteintech (Wuhan, China) and used in a 1:400 dilution. The Alexa555-conjugated phalloidin was purchased from Life iLab Bio-Technology (AC18L022; Shanghai, China) and used in a 1:1,000 dilution.

### Statistical Analysis

The results have been collected from at least three independent experiments. Data are expressed as the mean ± standard error (SE) and compared across groups by the one and two-way ANOVA with Dunnett’s *post hoc* test or 2-tailed Student’s t-test. For electrophysiological normalized data analysis, a Wilcoxon Mann–Whitney U test was used to compare currents with the drug to control currents. A *p*-value < 0.05 was considered statistically significant. GraphPad Prism (version 9.2) and Origin (version 7.5) were used for computations and graphing.

## Results

### Chronic Administration of Fluvoxamine and Lopinavir Produced a More Severe Inhibitory Effect on the Human Ether‐à‐go‐go–Related Gene Channel

**FIGURE 1:**
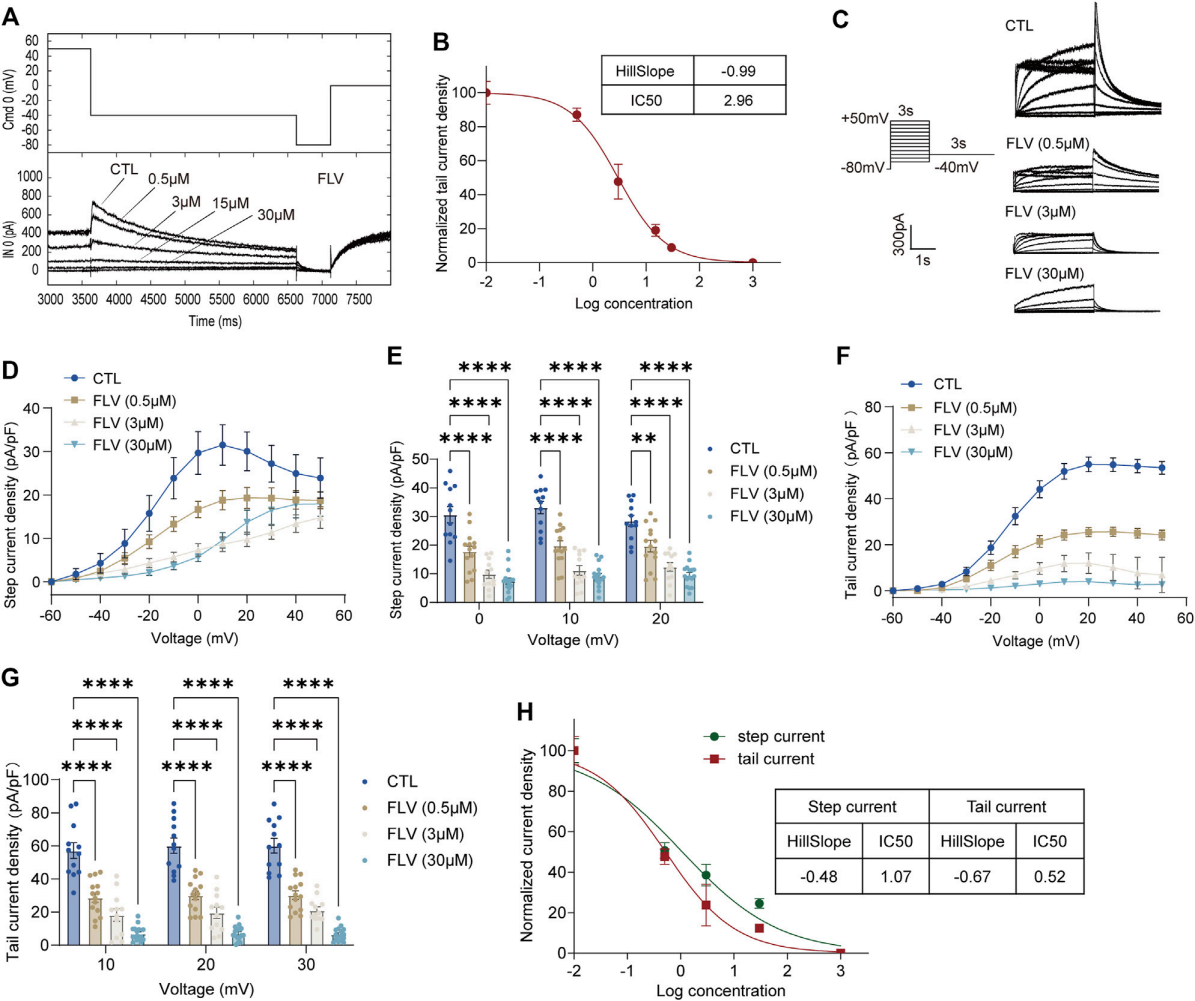
Inhibition of the hERG currents by fluvoxamine (FLV). **(A)** Concentration-dependent acute inhibition of hERG tail currents elicited by the protocol is shown. **(B)** Normalized data of tail current density at + 50 mV were fitted with the Hill equation to yield a concentration for half-maximal inhibition (IC50) and a Hill coefficient, *n* = 5. **(C)** Representative I_hERG_ recorded in control (CTL) and after drug application for 24 h with the voltage protocol. **(D)** Original data using the membrane capacitance (pF) of each cell to calculate the step current density (pA/pF) to form the current I–V curve of the hERG channel. **(E)** Bar chart showing statistical step current at 0 mV to + 20 mV in CTL or FLV with different concentrations. **(F)** I–V curve of the tail current density (pA/pF). **(G)** Statistical tail current density at + 10 mV to + 30 mV, *n* = 12–16. **p < 0.01 and ****p < 0.0001. **(H)** Normalized data on step and tail current density at + 30 mV were fitted with the Hill equation yielding an IC50 and a Hill coefficient.

**FIGURE 2:**
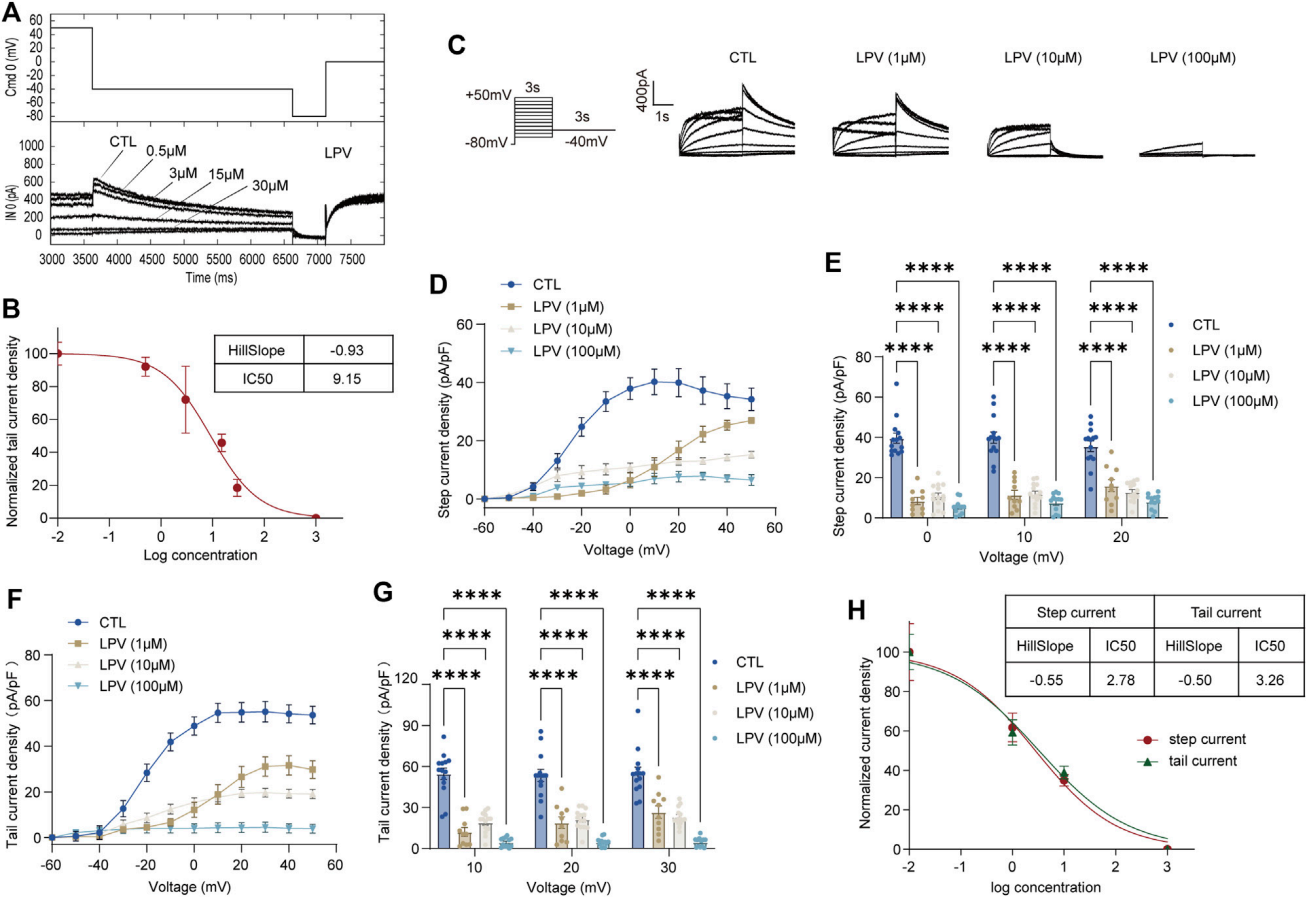
Inhibition of the hERG currents by lopinavir (LPV). **(A)** Concentration-dependent acute inhibition of hERG tail currents elicited by the protocol is shown. **(B)** Normalized data of tail current density at + 50 mV were fitted with the Hill equation to yield a concentration for half-maximal inhibition (IC50) and a Hill coefficient, *n* = 5. **(C)** Whole-cell patch-clamp for representative I_hERG_ recorded in control (CTL) and after LPV (1–100 μM) for 24 h of treatment recorded by the voltage protocol. **(D)** Original data using the membrane capacitance (pF) of each cell to calculate the step current density (pA/pF) to form the I–V curve of the hERG channel. **(E)** Corresponding bar chart shows the statistical step current at 0 mV to + 20 mV. **(F)** I–V curve of tail current density (pA/pF). **(G)** Statistical tail current density at + 10 mV to + 30 mV; *n* = 12–16. ****p < 0.0001. **(H)** Data on step and tail current density at +30mV were fitted with the Hill equation yielding an IC50 and a Hill coefficient.

To compare the inhibition of hERG channels at different drug application times, we initially used a series of concentrations ranging from 0.5 to 30 μM of FLV and LPV for acute perfusion of HEK-293 cells (Figure 1A and Figure 2A). The hERG current (I_hERG_) in the per-cell was recorded within 2 min for assessing IC50. The fitted data of relevant dose-dependent inhibitory curves were obtained by nonlinear regression fitting**.** We found that FLV and LPV acutely blocked I_hERG_ at the voltage condition of + 50 mV with an IC50 of 2.96 μM and 9.15 μM, respectively (Figure 1B and Figure 2B). For the long-term inhibitory effect of FLV and LPV on I_hERG_, drugs were incubated at least 24 h. The I_hERG_ was recorded in cells grown in the absence of drugs (CTL in Figure 1C and Figure 2C) or after FLV incubation (Figure 1C) or LPV incubation (Figure 2C), respectively. Statistical analysis demonstrated that FLV changes the step current density from 31.52 pA/pF at CTL to 9.61 pA/pF at 30 μM at the maximum activation voltage of 10 mV (Figure 1D,E). For LPV, the result showed that it exerted a more serious pharmacologically blockade from 40.19 pA/pF to 11.78 pA/pF at 10 μM at the 10 mV (Figure 2D,E). To examine the effects of FLV and LPV on a typical tail current of the hERG channel under the same concentration group, the peak tail current was also evaluated. Statistics showed a 10-fold reduction after maximal FLV concentration of 30 μM (Figure 1F,G) and a 13-fold reduction after 24-h treatment of maximal LPV concentration of 100 μM (Figure 2F,G), respectively. In total, 12–16 cells of I_hERG_ under each concentration were examined.

The IC50 at the voltage condition of + 30 mV was generated at fitted current values. They revealed that long-term treatment of drugs generates a lower IC50. FLV exerted a blocking effect in the step current with IC50 of 1.07 μM with the 95% confidence interval (95% CI) of 0.62–1.86 μM and the Hill slope (Hill coefficient) is −0.48, and that in the tail current with 0.52 μM with 95% CI of 0.28–0.95 μM along with a Hill slope of -0.67 (Figure 1H). Also, the observed IC50 for the LPV-inhibited step and tail current is 2.78 μM with the 95% CI of 1.31–5.88 μM and 3.26 μM with a 95% CI of 1.66–6.42 μM, respectively. The Hill coefficients for the fit of step and tail current are −0.55 and −0.50, respectively (Figure 2H). Of note, chronic drug incubation yields a Hill slope of a lower value relative to the acute effect, suggesting that acute drug applications can bind directly and block the channel even at lower concentrations.

### Fluvoxamine and Lopinavir Left-Shift the Activation Curve and Slow Activation of the Effect on the Human Ether‐à‐go‐go–Related Gene Channel

To investigate the activation-voltage gating of the hERG channel, the current–voltage relationships from the normalized peak tail current were fitted to the Boltzmann equation to obtain the half-maximal activation voltages (V_1/2_) and slope of the relationship curve K value representing the speed of the channel activation.

**FIGURE 3:**
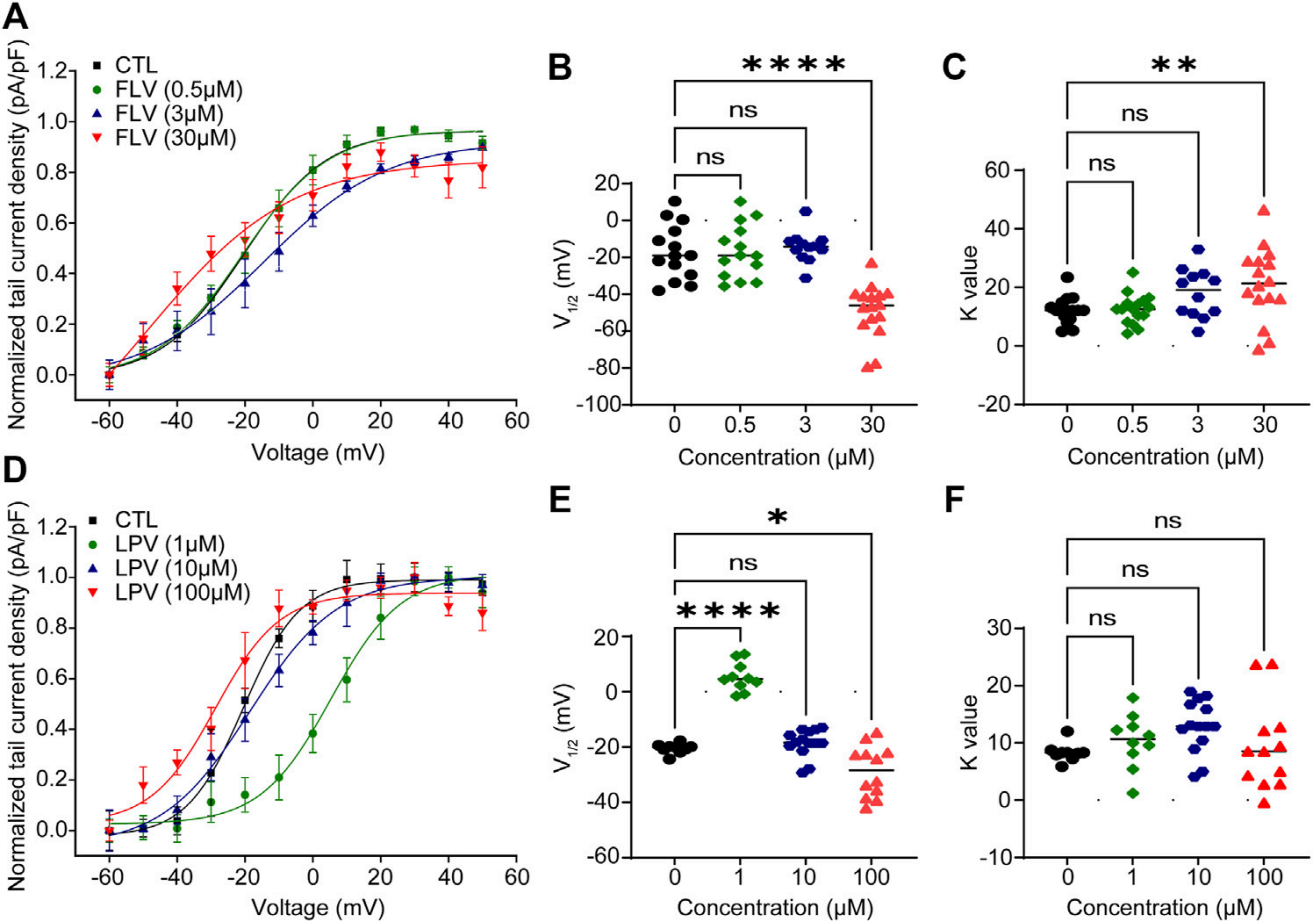
FLV and LPV cause left-shifting of the voltage-dependent activation curve and dramatic slowing of activation. **(A,D)** Peak tail current amplitude is normalized to their respective maximal amplitude and using the current density (pA/pF) of each cell to form the fitted peak tail current I–V curve by the Boltzmann function to obtain the voltage for half-maximal channel activation (V_1/2_) and slope factors K value from the curve of control (CTL) or each inhibitory concentration. **(B,C)** Scatter plots of individual values showing V_1/2_ and K values at 0 μM (CTL) to 30 μM FLV (*n* = 12–16). **p < 0.01 and ****p < 0.0001 compared with CTL. **(E,F)** Scatter plots of individual values showing V_1/2_ and K values at 0 μM (CTL) to 100 μM LPV (*n* = 9–14). *p < 0.05 and ****p < 0.0001. ns, non-significant.

The fitting results are summarized in Figure 3. Under the action of the lowest concentration of 0.5 μM of FLV, an activation curve almost overlapping with control (CTL) was generated (V_1/2_: 20.40 ± 1.31 mV in CTL; −20.65 ± 1.50 mV in 0.5 μM. K: 12.18 ± 1.19 in CTL; 12.59 ± 1.36 in 0.5 μM, *n* = 14) (Figure 3A). In the highest concentration of 30 μM, FLV left-shifted the V_1/2_ to negative voltages by 28.18 mV (−48.58 ± 17.98 mV in 30 μM, *p* < 0.0001) (Figure 3B) and increased the slope factor K value by 8.98 (21.16 ± 7.29 in 30 μM, *p* < 0.01, *n* = 16) (Figure 3C). For LPV, although a larger positive voltage of V_1/2_ was observed at the concentration of 1 μM, similarly, 100 μM LPV left-shifted the V_1/2_ of CTL (−20.53 ± 0.61 mV, *p* < 0.05) to −28.74 ± 2.67 mV (Figures 3D,E) and induced a negative alteration in the slope factor (8.33 ± 0.54 in CTL; 9.46 ± 2.24 in 100 μM, *n* = 9–14) (Figure 3F). These fitted data suggest that higher concentrations of FLV and LPV alter the activation gating of hERG channels, specifically exhibiting a leftward shift and an increased slope of the curve. This alteration was not observed in the low concentration group, indicating that higher doses of drugs were able to bind channels faster to decrease channel opening time and maintain a slow activation rate.

### 24-h Incubation of Fluvoxamine and Lopinavir Promotes the Intracellular Trafficking of the Human Ether‐à‐go‐go–Related Gene Protein

The inhibition of I_hERG_ is generally considered to be a direct channel block or a disruption of the channel protein trafficking by multiple groups of drugs (Zequn et al., 2021). Following nuclear transcription, a nascent peptide of hERG undergoes preliminary processing and core-glycosylation in the endoplasmic reticulum to form an immature precursor protein (135 kDa), and further glycosylation in the Golgi apparatus to form a mature protein (155 KDa) generating currents (Ficker et al., 2003; Li et al., 2011; Foo et al., 2016; Ono et al., 2020).

**FIGURE 4:**
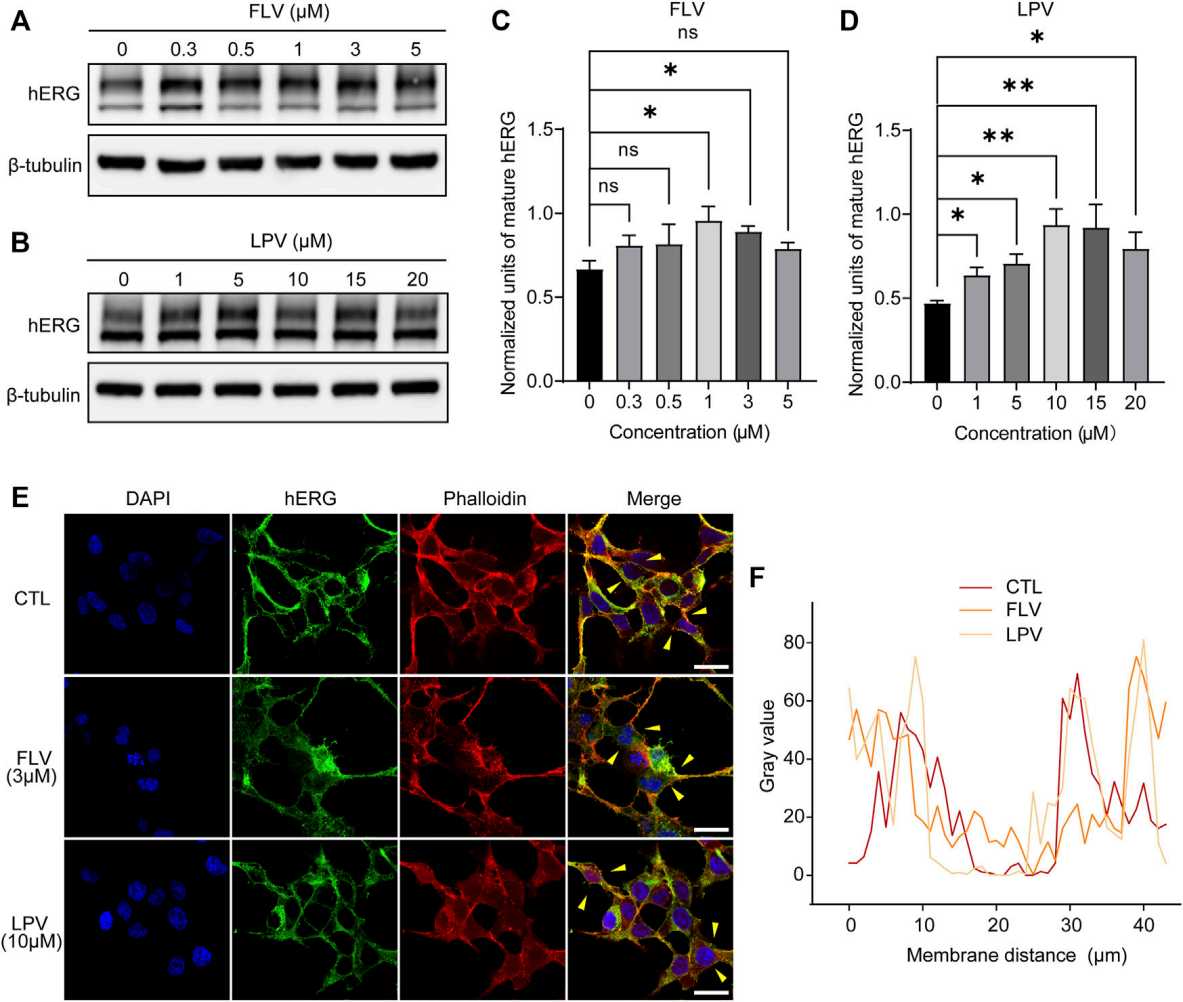
FLV and LPV facilitate the intracellular maturation of the hERG protein. **(A,B)** Western blot of mature (155KDa, above) and immature (135KDa, below) hERG protein after 24 h of incubation with FLV from 0.3 to 5 μM or LPV from 1 to 20 μM, respectively, along with internal control β-tubulin. **(C,D)** Densitometric evaluations of normalized mature hERG. **(E,F)** Immunostaining images of HEK-hERG stained with hERG (green), F-actinin (red, phalloidin), and DAPI (blue) before (CTL) and after 24 h of 3 μM FLV and 10 μM LPV treatment. Yellow arrowheads represent the area showing plasma membrane hERG staining. Scale bar: 10 µm. The levels of hERG without significant change at the membrane level after drug application are noted through a line scan plot showing co-localizing intensities of the hERG and F-actinin.

To examine whether the decrease in I_hERG_ is due to the suppression of the maturation of hERG proteins, we used Western blotting to detect the different forms of hERG proteins in the presence or absence of drug treatment. Unexpectedly, the results revealed that compared with the control group (0 μM), a mature form of the hERG channel protein increased under the action of FLV in the concentration range of 0.3–5 μM (Figure 4A), with statistical significance at 1 μM and 3 μM but no significance at 5 μM (Figure 4C). Similarly, LPV from 1 μM to 20 μM also significantly promoted the maturation of the hERG protein, particularly at the 10 μM and 15 μM (Figure 4B,D). The data were standardized with internal control β-tubulin and represented the mean of three experiments. Note that in the aforementioned concentration groups promoting hERG maturation, I_hERG_ is all reduced. For further intracellular evidence, confocal images of the hERG protein were used to detect its cellular localization. We focused on the co-localization of the hERG protein (green fluorescence) in the outermost periphery of the cell cytoskeletal protein F-actinin (red fluorescence dyed with phalloidin), which roughly represents the expression level of mature hERG on the cell membrane (Figure 4E). The line scan plot of fluorescence density analysis showed that the membrane expression of hERG did not change significantly after FLV and LPV treatment compared with the control group (CTL) **(** Figure 4F **)**.

These opposite results suggest that the inhibition of I_hERG_ induced by FLV or LPV is not due to the disruption of intracellular trafficking of the hERG protein. Conversely, the increased expression of mature hERG indicates that the drugs may generate a serious directly blocking effect for the channel, thereby affecting ion penetration or gating kinetics to reduce I_hERG_.

### Fluvoxamine and Lopinavir Upregulate the Expression of Hsp70 and Knockdown of Hsp70 and Reverse Their Effect on the Human Ether‐à‐go‐go–Related Gene

**FIGURE 5:**
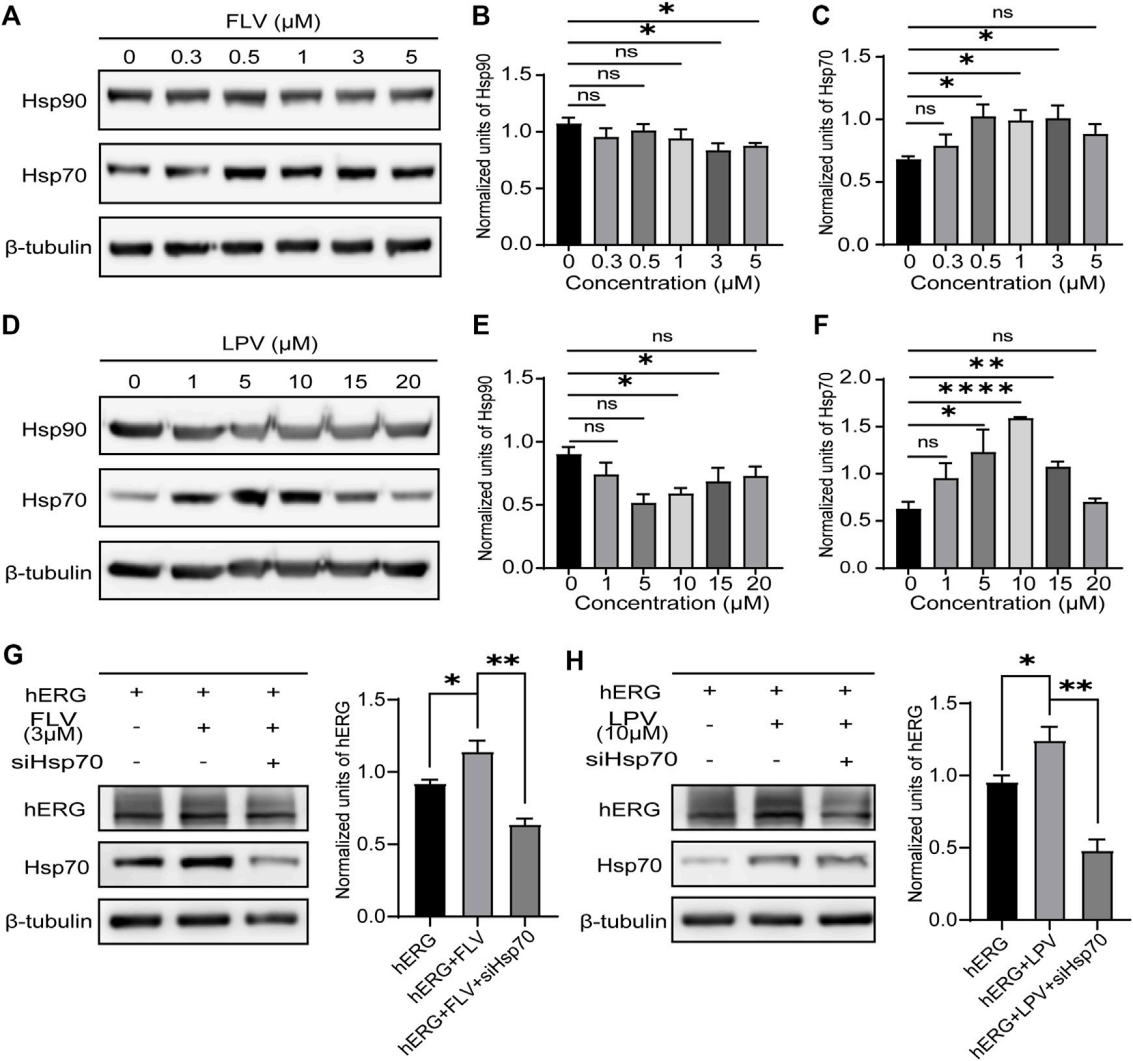
Reduced expression of Hsp70 reverses the role of FLV and LPV in promoting hERG maturation. **(A)** Western blot of critical hERG trafficking chaperone Hsp90 and Hsp70 in control (0 μM) and 24 h incubation with FLV from 0.3 to 5μM, along with internal control β-tubulin. **(B,C)** Densitometric evaluations of Hsp90 and Hsp70 normalized with β-tubulin under each drug concentration, respectively. *p < 0.05. ns, non-significant. **(D)** Western blot of Hsp90 and Hsp70 protein in control and FLV from 1 to 20μM, along with internal control β-tubulin. **(E,F)** Densitometric evaluations of normalized Hsp90 and Hsp70. **(G)** Immunoblotting and band density statistics for hERG, Hsp70, and the internal reference β-tubulin before (hERG) and after 24 h incubation of FLV with (hERG + FLV + siHSP70) and without (hERG + FLV) the reduced expression of Hsp70 using siRNA. **(H)** Immunoblotting and band density statistics for hERG, Hsp70, and the internal reference β-tubulin in the hERG, hERG + LPV, and hERG + LPV + siHSP70 groups. *p < 0.05, **p < 0.01, and ****p < 0.0001. ns, non-significant.

Previous studies have confirmed that Hsp90 and Hsp70 of the heat shock family located in the cytoplasm play a significant role in the intracellular quality control of hERG protein trafficking. The reduced expression and inhibition of Hsp90 and Hsp70 will significantly decrease the maturation of the hERG protein and accelerate the degradation of the immature protein (Ficker et al., 2003; Li et al., 2011). Given the critical role of these chaperone proteins, we investigated the chronic effect of FLV and LPV on these chaperones in cells. As expected, FLV and LPV significantly increased the expression of Hsp70, especially LPV at 10 μM (Figures 5A,C,D and F). In contrast, there was a significant reduction in the expression levels of Hsp90 after drug application (Figures 5A,B,D,E). These results may attribute the increase in the hERG expression induced by FLV and LPV to their altered expression of molecular chaperones.

To further determine whether the drugs increase the hERG expression by inducing a high expression of Hsp70, we used ideal siRNAs to knockdown Hsp70 after drug action. Expectedly, we found that knockdown of Hsp70 significantly reduced hERG maturation in the presence of FLV (Figure 5G) and LPV (Figure 5H). These findings suggest an important role for Hsp70 in the promotion of the hERG protein by FLV and LPV.

### Fluvoxamine and Lopinavir Shorten the Action Potential Duration and Induce Early After-Depolarization in Human-Induced Pluripotent Stem Cell–Derived Cardiomyocytes

**FIGURE 6:**
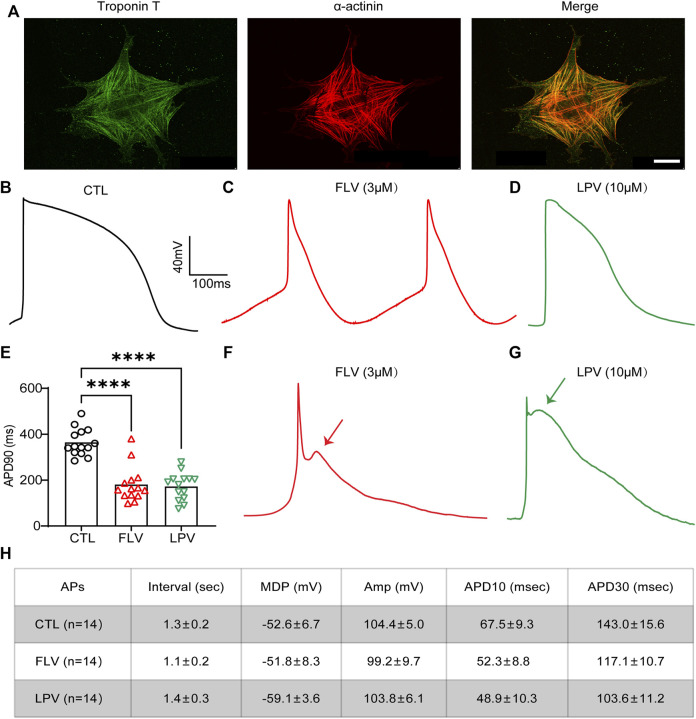
FLV and LPV shorten the action potential duration (APD) and induce early after-depolarizations (EADs) in the human-induced pluripotent stem cell–derived cardiomyocytes (hiPSC-CMs). **(A)** Immunostaining images of the specific cardiomyocyte markers troponin T (green) and α-actinin (red) to characterize the generation of hiPSC-CMs. Scale bar: 50 µm. **(B,C,D)** Representative action potentials (APs) in the absence of (CTL) and after 24 h of FLV (spontaneous APs) and LPV (evoked APs) incubation. **(E)** Statistical APD90 of 24-h FLV and LPV incubation compared with CTL, respectively, n = 14. **(F,G)** Representative APs after transient effects (within 1 hour) of FLV and LPV, respectively. Arrows represent the induction of the EADs. **(H)** Text for details of action potentials (APs). Interval, spontaneous rate interval; MDP, maximum diastolic potential; Amp, amplitude; APD, AP duration at different levels of repolarization.

Although still phenotypically immature, hiPSC-CMs possess a relatively complete ion channel composition and can better replicate cardiac electrophysiological activity *in vitro* (Ma et al., 2011; Ahmed et al., 2020). It represents an ideal and unique platform to test novel prospective therapeutic compounds or cardiotoxic drugs. Here, hiPSC-CMs generated from hiPSC using the directional differentiation reagent were confirmed by expressing cardiac-specific genes troponin T and a-actinin positive sarcomeric striations (Figure 6A). These iPSC-CMs were able to be used for study after day 14.

To assess the effects of FLV and LPV on the APD in hiPSC-CMs by inhibiting I_Kr_, 3 μM FLV and 10 μM LPV were used to treat cells. After 24-h of incubation, a whole-cell patch-clamp was used to record action potentials (APs) and ventricular-like APs with a distinct plateau phase (phase 2) after which repolarization accelerates (phase 3) were used for analysis. When contrasting the drug-treated groups with the control group (CTL) (Figure 6B), we did not observe a drug-induced prolonged APD but a significantly shortened APD in hiPSC-CMs (Figure 6C,D). Statistics showed that FLV and LPV decreased APD by 183.6 and 191.8 ms, respectively (364.4 ms in CTL; 180.8 ms in 3 μM FLV; 172.6 ms in 10 μM LPV. *p* < 0.0001, *n* = 14). (Figure 6E). To exclude the interference of protein production, cells treated with the same concentration were recorded within 1 hour to examine the transient effects of the medicines on hiPSC-CM_S_. Surprisingly, early after-depolarizations (EADs) during action potential repolarization were detected following drug treatment (Figure 6F,G). Together, our findings show that FLV and LPV can induce the occurrence of EADs, while the chronic effect shortens APD, implying that these drugs may affect other ion channels in hiPSC-CMs.

### Fluvoxamine and Lopinavir Have Strong Activity on Cardiac Ion Channels

**FIGURE 7:**
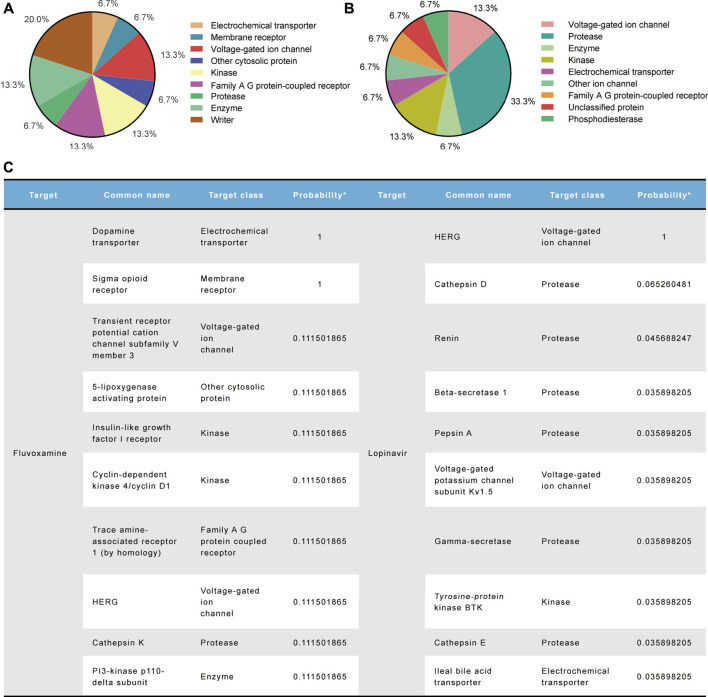
Molecular target prediction for FLV and LPV. **(A,B)** Pie charts summarize the predicted target classes for the top 15%. **(C)** Top 10 ranked target molecules for both compounds according to the probability values.

Following drug treatment, as we observed an unexpected shortening of the APD in hiPSC-CMs, we believed that the cardiac ionic currents, especially the inward currents, were similarly disturbed by them. We used the SwissTargetPrediction database to identify molecular-relevant targets for compounds. Target classes were summarized in a pie chart representing the top 15 of all predicted targets (Figure 7A and B). Both compounds had high activity on voltage-gated ion channels. Also, the top 10 target molecules showed hERG as their prominent target, especially LPV, with a probability value of 1 representing a known active (Figure 7C). Moreover, we did not observe them being active for inward calcium currents (probability value 0 in FLV, not predicted in LPV), and for sodium currents, probability value 0.1 in FLV and 0 in LPV (data not shown). Notably, in addition to the hERG potassium channel, a non-selective ion channel TRPV3 with a probability value of 0.1 and a potassium channel KCNA5 accompanied by a probability value of 0.03 were predicted in the FLV and LPV, respectively (Figure 7C). Together, although inward currents were not listed as significant targets for the aforementioned compounds, their substantial activity on cardiac voltage-gated ion channels still poses a challenge to explain the drug-induced changes in action potential duration.

### Fluvoxamine and Lopinavir Inhibit the L-Type Voltage–Operated Calcium Channel

**FIGURE 8:**
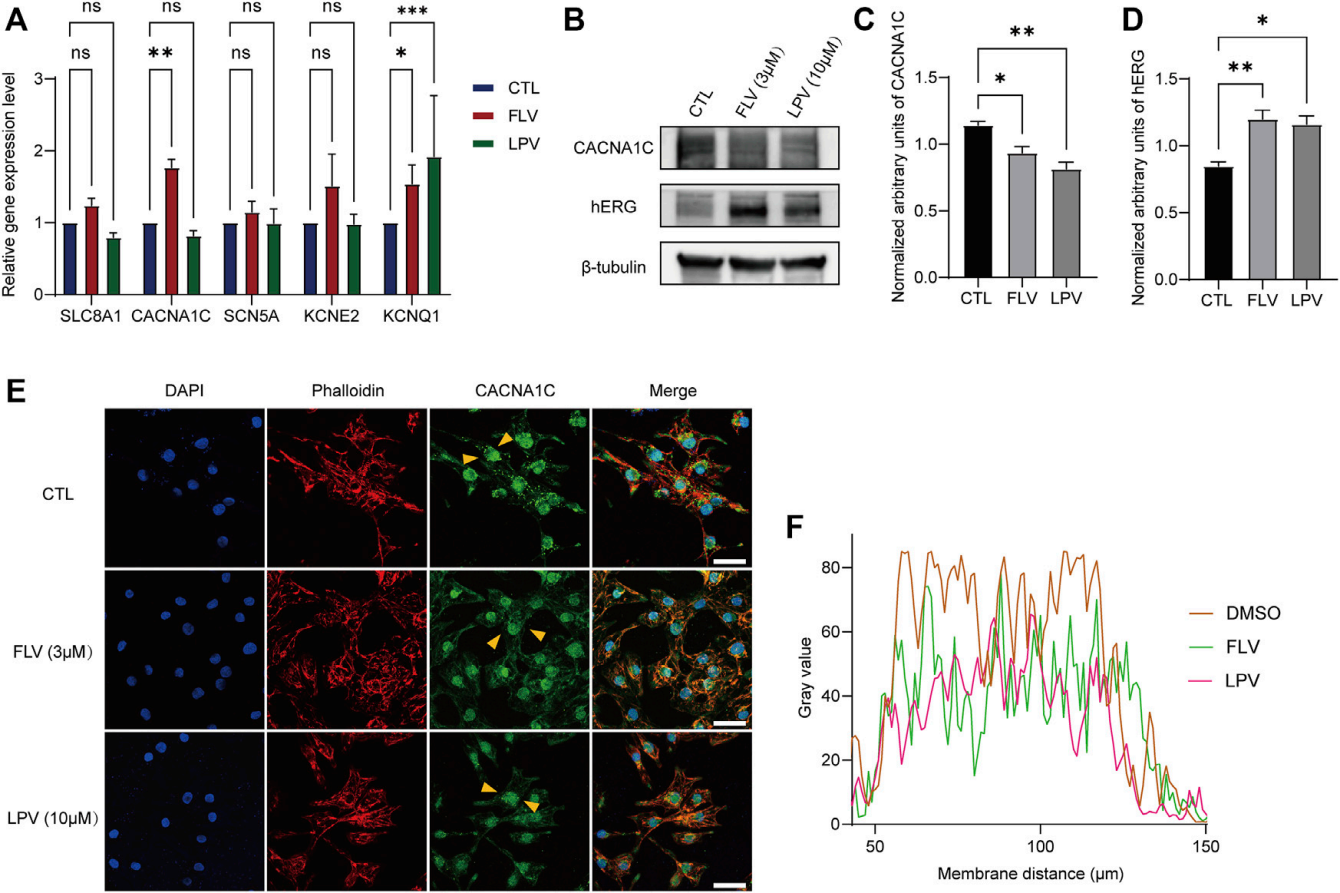
FLV and LPV inhibit L-type calcium channels. **(A)** Relative nucleic acid expression levels of cardiac ion channels determined by real-time quantitative PCR (RT-qPCR) with GAPDH as the housekeeping gene. **(B)** Immunoblotting of L-type calcium channel protein Cav1.2 (CACNA1C) and hERG protein in the presence and absence of drug incubation, with β-tubulin as an internal reference. **(C,D)** Assessment of band densities of normalized CACNA1C and hERG, respectively. **(E,F)** Immunostaining of hiPSC-CMs stained with CACNA1C (green), F-actinin (red, phalloidin), and DAPI (blue) before (CTL) and after 72 h of 3 μM FLV and 10 μM LPV treatment. Yellow arrows represent typical areas of fluorescence intensity evaluation, as shown in a line scan plot. Scale bar: 10 µm.

To further explore the activity of the drug on other cardiac ion channels, we examined the expression levels of five genes encoding different ion channels in hiPSC-CMs. They are *SLC8A1* encoding the sodium/calcium exchanger, *CACNA1C* forming the L-VOCC Cav1.2, *SCN5A* encoding the Nav1.5 channel, *KCNE2* that forms the hERG accessory subunit MinK-Related Peptide-1 (MiRP1), and *KCNQ1* encoding Kv7.1 to generate slow activation of delayed rectifier potassium currents (I_Ks_). RT-qPCR analysis showed that 3 μM FLV and 10 μM LPV did not significantly alter the mRNA expression of sodium or calcium channels (Figure 8A). However, we found that they significantly reduced the Cav1.2 protein expression; similarly, in cardiac myocytes, FLV and LPV increased the expression levels of the hERG protein (Figures 8B–D). Subsequently, we detected the intracellular expression of Cav1.2 after drug incubation by confocal microscopy. Compared to DMSO-treated hiPSC-CMs, FLV and LPV-treated cells had a lower calcium channel fluorescence density (Figure 8E,F). These findings suggest that although the nucleic acid levels of Cav1.2 were not altered, the low expression of the protein suggests the inhibitory activity of the COVID-19 drug on L-VOCC.

## Discussion

In this study, we investigated the chronic effects of the COVID-19 drugs FLV and LPV on the cardiac primary repolarizing potassium current, I_Kr_. Combining molecular experiments and electrophysiological analysis, our data showed that a long-term incubation of FLV and LPV blocked I_hERG_ of step current and peak tail current more severely with a lower IC50. Also, they left-shifted the activation curve of the channel as well as slowed its activation rate, as evidenced by the increased slope factor K value. FLV and LPV did not exert inhibition on the hERG protein expression but promoted its maturation, and caused an upregulated level of Hsp70. Knockdown of Hsp70 reverses drug-induced maturation of the hERG protein. Moreover, FLV and LPV induced a shortening APD in hiPSC-CMs and the occurrence of EADs by transient treatments.

Our results showed that FLV and LPV produced a sustained inhibition of the hERG channel, an effect that was more severe than the acute treatment, manifested by a lower IC50 and newly characterized gating data. The work by Milnes et al. (2003) showed an acute reduction of the hERG current by FLV at an IC50 value of 3.8 μM and resulted in a leftward shift of the activation curve at a concentration of 3 μM. Chronic drug administration here produces a lower IC50 value and caused altered channel gating at higher concentrations of 30 μM. For LPV, Anson et al. (2005) demonstrated that it inhibited the hERG channel in a concentration-dependent manner with an IC50 of 8.6 μM and reduced I_Kr_ by 40% at 10 μM; in our study, chronic incubation at 10 μM reduced the control from 40.19 pA/pF to 11.78 pA/pF. Moreover, FLV and LPV left-shifted the hERG channel activation curve and increased its K value in response to high concentrations, which could be responsible for FLV and LPV producing a relatively low IC50. Direct drug binding to hERG channel drug-sensitive sites to alter channel gating kinetics has been commonly reported (Sănchez-Chapula et al., 2003; Duan et al., 2007; Jo et al., 2008; Kan et al., 2017; Cheng et al., 2019). Cryo-EM structures of the hERG channel show that these drug-sensitive residues are localized to the four elongated hydrophobic pockets that form the selectivity filter (SF) (Wang and MacKinnon, 2017). A recent *in silico* study showed that several antivirals, including LPV, interact with the classical binding site at the base of the SF (Al-Moubarak et al., 2021). The SF at the pore domain (PD) plays an essential role in the activation and inactivation of the channel due to the presence of a voltage-sensing domain (VSD)/PD coupling and an activation gate (AG)/SF coupling (Zequn and Jiangfang, 2021). Thus drug binding at F656 and Y652 appears to frequently influence the activation or inactivation of the channel (Sănchez-Chapula et al., 2003; Duan et al., 2007; Jo et al., 2008; Kan et al., 2017; Cheng et al., 2019). As with acute effects, chronic incubation with FLV also left-shifted the activation profile of the channel. Despite the instability of hERG channel proteins on membranes rescued or inhibited by drugs, this alteration in gating kinetics could hardly be explained by changes in the expression of hERG proteins or molecular chaperones, but rather by drug binding sites causing gating-dependent changes.

Typically, the therapeutic dose of FLV in psychiatric disorders is 160–220 μg/L (0.38–0.51 μM), and we note that in RCT using FLV to treat COVID-19, a dose of 100 mg is used for treatment (Lenze et al., 2020). Although the pharmacokinetics of FLV was not tested, and even without considering the reduced plasma concentration associated with the first-pass elimination, we are still unable to determine that the concentration used does not affect the QT interval in COVID-19 patients. For LPV, usually used in combination with its synergist ritonavir and at a dose of 400mg/100 mg twice a day, the same dose was used to suppress the COVID-19 pandemic (Cao et al., 2020a). Fortunately, the research work has been conducted to show that LPV produces a 50% effective inhibition concentration (EC50) of 1.74 μM against SARS-CoV-2 (Lou et al., 2021), a value very close to our data for IC50 in I_hERG_. Also, note that we have obtained the data without the combination of ritonavir. These data highlight the importance of properly prescribing these medications and monitoring QT intervals because reliable information on drug concentrations or doses for treating COVID-19 is limited.

Molecular mechanisms underlying this inhibition show that even if the IC50 was increased 10-fold, instead of the decreased expression, FLV and LPV increase matured form of the hERG channel protein along with an upregulated Hsp70 and downregulated Hsp90. It helped us rule out the possibility that FLV and LPV inhibit hERG protein maturation in a concentration-dependent manner to cause defective channel currents. In general, Hsp90 acts downstream of Hsp70 to take over Hsp70-modified folding intermediates (Morán Luengo et al., 2019). Unlike Hsp/c70, which keeps most freshly generated proteins in a competent folding state, Hsp90 is essential for the folding of a small group of proteins that have trouble reaching their native conformations (Nathan et al., 1997). As a result, mutant hERG channel proteins may require more Hsp90 than wild-type hERG channel proteins. Combined with previous evidence (Ficker et al., 2003), the high expression of Hsp70 here may be responsible for the increased full glycosylation of hERG. Unexpectedly, Hsp90, a chaperone also critical for hERG maturation, was reduced. Although reduced Hsp90 expression has not been linked to increased hERG expression, medicines targeting the hERG channel inhibit Hsp90 (Zhang et al., 2014; Yu et al., 2017). Importantly, Hsp90 inhibitors also upregulate other heat shock proteins such as Hsp70 by dissociating HSF1 from the Hsp90-HSF1 complex to enhance its transcriptional activity, which is critical for cellular homeostasis (Sha et al., 2017; Morán Luengo et al., 2019; Hintelmann et al., 2020; Chaudhury et al., 2021). Further experiments on compound–protein interactions are required to qualify these drugs as Hsp90-targeted inhibitors. Although a variety of drugs have been shown to interfere with hERG transport to reduce current, direct blockade of drugs without trafficking defects has also been reported (Borsini et al., 2012; Tanaka et al., 2014). Recently, a study of the effects of COVID-19 drugs chloroquine and hydroxychloroquine on blocking the hERG potassium channel showed that these drugs acutely and severely inhibited the hERG current, but remdesivir increased the I_hERG_ with promoted hERG maturation when acting alone (Szendrey et al., 2021). Interestingly, chloroquine prompted the trafficking of the hERG protein (Borsini et al., 2012). Combined with our results, the opposite trend of protein expression and current inhibition suggested that FLV and LPV produced distinctive hERG channel blocking mechanisms. Also, it can be concluded that the increase in the hERG expression may be attributable to drug-induced upregulation of Hsp70 and compensatory downregulation of Hsp90, as the direct binding action of these medicines on the hERG channel significantly surpasses the level at which they stimulate mature protein production under the chronic action.

We also used hiPSC-CMs to evaluate the long-term effect on FLV and LPV. Unexpectedly, the hERG inhibition–induced prolongation of APD was not observed here; instead, FLV and LPV shortened APD. Different phenotypes caused by drugs in cardiomyocytes and heterologous expression systems can be explored as follows: first, in contrast to hiPSC-CMs, HEK-hERG simply and roughly replicates the major *α* subunit of the I_kr_ channel, regardless of the interaction of other ion currents (Shah, 2005). Second, APD in hiPSC-CMs containing various ion channels is shortened by FLV and LPV treatments, implying that if these drugs produce inhibition of I_kr_, their effect on other inward ion currents, particularly the L-type calcium current (I_CaL_) in the plateau phase should be more pronounced. Theoretically, a decrease in outward ion currents such as hERG current and an increase in inward ion currents such as sodium and calcium currents in the heart would lead to a prolongation of the QT interval (Vicente et al., 2019). When paired with antimalarials that target the hERG channel also acting on a variety of inward ion currents including I_CaL_ and at higher concentrations, COVID-19 drugs may interfere with I_Ks_, I_K1_, and/or transient outward potassium current (I_to_) potassium current, and even inhibit sodium and I_CaL_ current (Sánchez-Chapula et al., 2001; Cubeddu et al., 2022). Indeed, in the final experiments, we found that chronic FLV and LPV administration increased the level of *KCNQ1* nucleic acid producing I_Ks_ and decreased the expression of CNCNA1C calcium channel protein. Importantly, as a sigma-1 receptor agonist, FLV enhances I_to_ and decreases I_CaL_ (Chen et al., 2020; Guo et al., 2021). The enhancement of another outward current and the suppression of the inward current may exceed the effect of the drug-induced hERG inhibition, thus shortening the APD. Further isolation of these ionic currents in hiPSC-CMs in the presence of drugs can provide additional evidence to support this.

Together, as an unfavorable effect, drug-induced hERG block and potential LQTS represent a significant concern for drug safety (Inciardi et al., 2020). Related drugs in COVID-19 lead to prolonged QT intervals, and even sudden cardiac death has been reported (Garcia-Cremades et al., 2020; Sun et al., 2020; Szekely et al., 2020). Our data show that in the absence of hERG maturation decrease, the long-term administration of FLV and LPV generates a more drastic impact on the reduction of I_hERG_, which provides a newly single molecular insight into understanding the drug-induced QT prolongation. It is worth noting that COVID-19 patients with pre-existing inherited arrhythmia syndromes receiving drugs that target the hERG channel are more likely to develop LQTS (Shah, 2005), thus monitoring ECG recordings for COVID-19 patients to prevent and treat arrhythmias promptly when receiving the appropriate drug treatment.

## Data Availability

The original contributions presented in the study are included in the article/Supplementary Material, further inquiries can be directed to the corresponding authors.

## Abbreviations

APD, action potential duration; COVID-19, novel coronavirus disease of 2019; EADs, early after-depolarizations; ECG, electrocardiogram; FLV, fluvoxamine; HEK-293, human embryonic kidney-293 cells; hERG, human ether‐à‐go‐go–related gene; hiPSC, human-induced pluripotent stem cell; hiPSC-CMs, human-induced pluripotent stem cell–derived cardiomyocytes; HSPs, heat shock proteins; IC50, half-maximal inhibitory concentration; ICaL, L-type calcium current; IhERG, hERG current; IKr, rapid activation of delayed rectifier potassium currents; IKs, slow activation of delayed rectifier potassium currents; Ito, transient outward potassium current; LPV, lopinavir; LPV/r, lopinavir/ritonavir; L-VOCC, L-type voltage–operated calcium channel; MiRP1, MinK-related peptide-1; PD, pore domain; RCT, randomized controlled trial; SARS-CoV-2, severe acute respiratory syndrome coronavirus 2; SF, selectivity filter; SRI, selective-serotonin reuptake inhibitor; VSD, voltage-sensing domain.
